# Epidemiology of Animal Poisonings in the Canary Islands (Spain) during the Period 2014–2021

**DOI:** 10.3390/toxics9100267

**Published:** 2021-10-14

**Authors:** Cristian Rial-Berriel, Andrea Acosta-Dacal, Manuel Zumbado, Luis Alberto Henríquez-Hernández, Ángel Rodríguez-Hernández, Ana Macías-Montes, Luis D. Boada, María del Mar Travieso-Aja, Beatriz Martin-Cruz, Alejandro Suárez-Pérez, Miguel Ángel Cabrera-Pérez, Octavio P. Luzardo

**Affiliations:** 1 Toxicology Unit, Research Institute of Biomedical and Health Sciences (IUIBS), Universidad de Las Palmas de Gran Canaria, Paseo Blas Cabrera s/n, 35016 Las Palmas de Gran Canaria, Spain; cristian.rial@ulpgc.es (C.R.-B.); andrea.acosta@ulpgc.es (A.A.-D.); manuel.zumbado@ulpgc.es (M.Z.); luis.henriquez@ulpgc.es (L.A.H.-H.); anrodrivet@gmail.com (Á.R.-H.); ana.macias@ulpgc.es (A.M.-M.); luis.boada@ulpgc.es (L.D.B.); beatriz.martin@ulpgc.es (B.M.-C.); 2 Study Group on Wild Animal Conservation Medicine (GEMAS), 28040 Madrid, Spain; 3 Spanish Biomedical Research Center in Physiopathology of Obesity and Nutrition (CIBERObn), 28029 Madrid, Spain; 4 Grupo Hospitalario San Roque, C/Dolores de la Rocha, 35001 Las Palmas de Gran Canaria, Spain; marimartravieso@gmail.com; 5 “La Tahonilla” Wildlife Recovery Center, 38111 Santa Cruz de Tenerife, Spain; alejandro.suarezperez@ulpgc.es; 6 General Directorate to Combat Climate Change and the Environment, Biodiversity Service, Canary Islands Government, Plaza de los Derechos Humanos, 35071 Las Palmas de Gran Canaria, Spain; mcabperd@gobiernodecanarias.org

**Keywords:** banned pesticides, intentional poisoning, carbofuran, aldicarb, anticoagulant rodenticides, QuEChERS, LC-MS/MS, GC-MS/MS

## Abstract

Animal poisoning is one of the greatest conservation threats facing wildlife. In a preliminary study in the oceanic archipelago of the Canary Islands, we showed that the degree of threat from this circumstance was very high-even higher than that reported in other regions of continental Europe. Consequently, a legal framework for the effective prosecution of the crime of wildlife poisoning came into force in 2014 in this region. We present the results of the investigation of 961 animals and 84 baits sent to our laboratory for the diagnosis of animal poisonings during the period 2014–2021. We were able to identify poison as the cause of death in 251 animals and 61 baits. Carbofuran stands out as the main agent used in this archipelago. We have also detected an increasing tendency to use mixtures of several pesticides in the preparation of baits. The entry into operation of two canine patrols has led to the detection of more dead animals in the wild and a greater number of poisoned animals. The percentage of poison positives is significantly higher in areas with lower population density, corresponding to rural environments, as well as in areas with greater agricultural and livestock activity.

## 1. Introduction

Poison remains one of the most important conservation threats faced by many wildlife species, and it also affects domestic animals frequently [1,2,3,4,5,6,7,8,9,10,11,12]. There are numerous chemicals that can affect animals, but perhaps the group of agricultural pesticides is the most important [13]. These compounds, commonly used and widespread in global food production, cause many accidental poisonings in non-target species [5,7,13,14], and accidental poisonings by these substances in humans are also frequently described [15,16]. Among the pesticides, rodenticides stand out, since they are directed against higher vertebrates-rodents-and can easily reach other non-target species that share their habitat [17]. Numerous studies have indicated that rodenticides can harm non-target species of mammals, reptiles, and birds, particularly birds of prey, but also other species, which do not necessarily feed on rodents or small mammals that have ingested the poison, but granivorous birds that directly ingest the baits [18]. Affected animals suffer anticoagulant and hemodynamic effects that predispose the animal to death [17,19,20,21,22]. Apart from pesticides, there are other substances that can poison wildlife, such as industrial pollutants or veterinary drugs. Like pesticides, veterinary drugs have the potential for bioaccumulation and transfer through food webs. Each year, several thousand tons of active ingredients are used in animal husbandry [18], and a portion of these may eventually result in environmental and ecological impacts [23]. Wildlife exposure to pharmaceuticals can occur through contaminated water [24], agricultural soils, plants, and arthropods [25,26,27], and through excreta and carcasses of medicated livestock (i.e., supplemental feeding of threatened scavenger birds) [28,29].

If accidental exposure to chemicals can cause harm to animal health, the situation becomes dramatic when chemicals are intentionally used in the preparation of baits to kill animals. Among the chemicals that baits may contain, pesticides, including rodenticides, are the most used [8,12,30]. It has been estimated that pesticides are used illegally in up to 68% of all suspected animal poisoning cases [1,5,7,14]. Due to their high toxicity, several restrictions have been applied to many compounds that are currently banned or severely restricted in the EU. However, these are not the only compounds intentionally used for the purpose of killing animals, and there is growing evidence that veterinary drugs and chemicals other than agricultural pesticides are being used for this purpose [31,32,33].

The Canary Islands, where this study has focused, constitute a Spanish archipelago located in the Atlantic Ocean off the northwest coast of Africa (between coordinates 27° 37′ and 29°25′ north latitude and 13°20′ and 18°10′ west longitude) consisting of eight inhabited islands and several uninhabited islets, all of them of volcanic origin. This archipelago is home to marine and terrestrial ecosystems of great value, both ecological and scenic, and represents a hotspot of biodiversity, with a huge number of endemic species, due to the evolutionary isolation from nearby continents (Africa and Europe). According to updated data, there are almost 4500 endemic species in the Canary Islands, representing more than 27% of the total biodiversity recorded (https://www.biodiversidadcanarias.es/biota/, accessed on 7 September 2021).

The archipelago has extensive areas of its territory with different levels of environmental protection (around 40% of its surface area), but it also has other areas subject to intense anthropogenic pressure, since it has a stable population of 2.1 million inhabitants and receives more than 12 million tourists a year. In such a small territory, poison baiting is particularly dramatic, as a single poisoning event can severely damage the populations of endemic species with only a few tens or hundreds of individuals, to the point of bringing them to the brink of extinction. Unfortunately, poisoning is a common practice in this archipelago, as could be documented in a first study that covered 4 years (2010–2013) and that aimed to highlight this dramatic problem [4]. As a consequence of this preliminary study, in 2014, the Strategy for the Prevention and Control of Poisoning in the Canary Islands was approved [34]. This law articulates a series of measures for research, public awareness, and prosecution of the crime of wildlife poisoning in this European region. This law names the Toxicology laboratory of the University of Las Palmas de Gran Canaria as the reference laboratory for the official investigation of poisoning incidents in this region, as it had been in charge of the official investigation of such events since January 2014 [4,34]. Consequently, our laboratory began to receive the totality of the samples generated in the incidents investigated by the environmental police and has been developing all the necessary methodology to perform the most complete search possible of all toxic substances that usually affect or could affect the health of wildlife and domestic fauna [35,36,37].

In this article, we present the description and epidemiological study of the results of the diagnostic service of wildlife poisonings that we provide to the Government of the Canary Islands in the period 2014–2021 (until June), including the results of 961 animals and 84 baits that have been investigated during this period.

## 2. Materials and Methods

### 2.1. Sampling

Blood and liver samples from 961 animals received in our laboratory for diagnosis of possible poisoning were analyzed between January 2014 and June 2021. During this period, we also received 84 meat baits. Occasionally, we also received other samples, such as gastric contents, degraded carcasses, cadaveric fauna, and insects collected at the scene of the incident. When necessary, these samples were used to clarify results found in blood or liver. However, none of the results we present were obtained solely from these ancillary samples, so cases where blood or liver were not available have not been included. It is noteworthy that 424 animals belong to species or subspecies endemic to the Canary Islands, highlighting 307 birds of prey and 74 lizards (*Gallotia* spp.), the latter being critically endangered. The animals correspond to a total of 753 incidents investigated, giving an average of 1.3 animals affected per incident (although they ranged from one to 20 animals per incident). The animals investigated belonged to 63 different species, including 44 dogs and 49 cats. Table 1 (Results and Discussion section) lists the species in which at least one chemical as the cause of death was detected. All animals were collected in the context of investigations of possible environmental crimes by environmental agents and were transferred to the wildlife rehabilitation centers of Tafira (in Gran Canaria) or La Tahonilla (in Tenerife) where liver samples for toxicological analysis were collected during necropsy. Once collected, the samples were kept frozen until they were transferred to the Clinical and Analytical Toxicology Service (SERTOX) of the University of Las Palmas de Gran Canaria (ULPGC, Canary Islands, Spain), where they remained frozen at −24 °C until their analysis. Most of the animals were found dead in the field or in urban areas or died while in veterinary facilities. No animals were sacrificed for the purposes of this study, and no experiments were performed on or with live animal samples.

### 2.2. Analytical Method

The methodology used for blood and liver samples allowed the search respectively for 360 and 351 compounds highly toxic to animals and has been fully validated according to international guidelines [38,39] and previously published by our group [3,35,36,40]. The complete list of analytes, as well as the technique used for their quantification, can be found in Appendix A. The extraction of blood and liver are based on the QuEChERS technique, although in our methods, a miniaturization of this technique has been performed, allowing the use of only 250 μL of blood [35,36] or 1 g of liver [3,40], without requiring any additional purification step, nor any change of solvent. For the rest of the samples (baits, gastric and intestinal contents, decomposing carcasses), a solid–liquid extraction was usually used, according to a procedure also previously published by our group [4,37], although in the case of these auxiliary samples, we consider the analyses to be semi-quantitative only.

The quantitative analysis was carried out in all cases by a combination of two complementary analyses, one by gas chromatography coupled to triple quadrupole mass spectrometry (GC-MS/MS) for the analysis of the more volatile compounds (mainly persistent organic pollutants and some less polar pesticides) and another analysis by liquid chromatography coupled to triple quadrupole mass spectrometry (LC-MS/MS) for pharmaceuticals, rodenticides, and more polar pesticides. For GC-MS/MS, an Agilent 7890B gas chromatograph (Agilent Technologies, Palo Alto, CA, USA) was used, which was equipped with two Agilent J&W HP-5MS (5% cross-linked phenyl-methyl-polysiloxane, Agilent Technologies) fused silica capillary columns, with a total length of 30 m (15 + 15 m), a film thickness of 0.25 μm, and 0.25 mm diameter each. The reason for using two columns joined by a purged joint was to allow the application of the backflushing technique that reduces background noise and prolongs the lifetime of the column. He 5.0 (99.999%) was used as the carrier gas, and N_2_ 6.0 (99.9999%) was used as the collision gas. Gases were from Linde (Dublin, Ireland). For LC-MS/MS, an Agilent 1290 Infinity II UHPLC instrument (Agilent Technologies, Palo Alto, CA, USA) was used. It was equipped with an InfinityLab Poroshell 120 column (2.1 mm × 100 mm, 2.7 µm), coupled to an in-line filter and a UHPLC guard column with the same characteristics as the analytical column. The detection and quantification of GC-separated compounds was performed with an Agilent 7010c mass spectrometer, and that of LC-separated compounds with an Agilent 6460 electrospray Jet Stream (AJS-ESI) mass spectrometer (Agilent Technologies, Palo Alto, CA, USA), which were both operated in dynamic multiple reaction monitoring (dMRM) mode, in positive (GC-MS/MS), and positive and negative (LC-MS/MS) polarities. All the technical details of the extractions, the programming of the equipment, and the results of the validation of the techniques can be found in the cited references [3,4,35,36,37].

All quantifications were performed against calibration curves prepared in matrix, blood, or liver as appropriate with a mixture of the 360 individual chemicals and the deuterated compounds used as procedural internal standards. All standards were of the highest purity available (93.1% to 99.8%) and purchased from various suppliers (A2S-Analytical Standard Solutions (Staint Jean D’Illac, France), Sigma-Aldrich (Augsburg, Germany), CPA Chem (Stara Zagora, Bulgaria), European Pharmacopoeia Reference Standards (Strasbourg, France), Accustandard (New Haven, CT, USA), and Dr. Ehrenstorfer (Augsburg, Germany)). The calibration curves were prepared with a minimum of 6 points, covering the range up to 2 μg/mL.

### 2.3. Statistical Analyses

All statistical analyses were performed with GraphPad Prism v9.2 software (GraphPad Software, San Diego, CA, USA). The distribution of the variables included in this study was evaluated using the Kolmogorov–Smirnov test. Given the nature of the cases investigated (animals due to poisoning), the concentrations of most of the chemicals detected did not follow a normal distribution, so the results presented in Table 2 are expressed in terms of median and range. For the same reason, we employed nonparametric tests to check for statistical differences between the concentrations found in the groups of animals, using the median rather than the mean. For the study of determinants of poisoning, the dichotomous categorization (0/1) was used for the absence or presence of substances in a concentration that was compatible with the poisoning of the animals, *regardless* of whether one or more substances were involved. The association study between the different determinants and the outcome (poisoning vs. non-poisoning) was evaluated with the Chi-square test (𝝌2). A *p*-value of less than 0.05 (two-tailed) was considered statistically significant in all the statistical analyses.

## 3. Results and Discussion

We studied the incidence of wildlife poisonings in the period from January 2014 to June 2021. We finally included liver or blood samples from 961 deceased animals and 84 baits. A total of 312 animals were referred with strong suspicion of poisoning, and of these, the presence of poisoning was confirmed in 223 animals (71.5%). The remaining 649 animals were sent to rule out the presence of poison, among other possible causes of death, and the poisoning was confirmed in 29 of them (4.5%). All baits were initially classified as intentionally supplied in the environment, and the presence of some type of poison was confirmed in 61 of them (72.6%). The total number of positive identifications represented 29.9% of the samples submitted during this period. The number of deceased animals in incidents where a chemical was detected in toxic concentrations was significantly higher than in negative cases (mean 2.53 ± 0.28 vs. 1.31 ± 0.15, *p* < 0.0001), as has been described in other works at the international level [6,7,40,41,42,43], and in the previous studies conducted in the Canary Islands [4,44].

In Table 1, we show the number of positive cases distributed by species and type of toxicants involved. The wildlife species with the highest number of positive cases was *Columba livia*, with 92.6% of the specimens submitted positive for poison (25/27). This can be explained by the fact that only pigeons for which a strong suspicion of poisoning had been received by our laboratory, in contrast with what occurred with other species. We also identified poisoning in a high number of *Falco tinnunculus canariensis* (32/108), *Gallotia* spp. (21/27), *Buteo buteo insularum* (27/52), *Neophron percnopterus majorensis* (17/49), *Asio otus canariensis* (22/84), *Corvus corax canariensis* (22/97), and *Pyrrhocorax pyrrhocorax barbatus* (7/10). These seven species or subspecies are endemic to the Canary Islands, and all of them are at a high degree of conservation threat, mainly due to human activities. Regarding domestic animals, we only received cases of dogs (26 positives out of 44 referrals) and cats (21 positives out of 49 referrals). In the previous study period (2010–2013) [4], these two species represented the highest percentage in the total diagnoses. In the period we now present, the percentage of positives among domestic animals has fallen appreciably, while the percentage of positives among wild species has remained fairly similar over the years [4].

Seventeen different toxicants were detected, and most of them were pesticides, except for amobarbital, which was identified as the probable cause of death in one case (*Corvus corax canariensis*). According to our results, in the positive cases, a mean of 1.54 ± 0.38 toxicants per incident were detected. The most frequently detected chemical was carbofuran, which was present in 97 animals and 24 baits (38.7% of positive cases). This percentage is very similar to that previously reported in the Canary Islands [4], which would indicate that the habit of using this compound to get rid of nuisance animals has not diminished in the Canary Islands. It should be noted that carbofuran has been banned in the EU since 2007. Almost 15 years after its withdrawal from the market, there still seems to be an important stock of this compound in private farms in the archipelago, which has not been handed over to the authorities and which continues to be used illegally. The use of aldicarb, also banned in the EU for almost two decades [45], does not seem to have decreased either, as it was detected in almost 10% of the positive cases (in the previous period, it was present in 12.2% of the positive cases [4]). In addition, the percentage of cases in which the cause of death was an anticoagulant rodenticide has remained practically unchanged between both periods (29.9% in 2014–2020 vs. 29.1% in 2010–2013). However, regarding the rest of the detected compounds, we did find differences between both periods, although this was not too important. We detected a greater variety of substances involved (17 vs. 14) as well as a greater involvement of other acetylcholinesterase inhibitor insecticides, such as chlorpyrifos, pirimiphos methyl, dimethoate, oxamyl, or methomyl. We were struck by the fact that unlike what happened in the previous study period, in these years, we have detected an increase in the cases in which several poisons are detected simultaneously, which became more evident in the study of baits. In 35.8% of the cases, the baits were prepared with between three and five different compounds, all of them commonly used in Canary Island agriculture in the past but mostly also substances currently banned in the EU. This could be due to the fact that stocks of the more potent poisons that have traditionally been used may be running low in some places, and poisoners seek to maximize the effectiveness of the baits they prepare by mixing different substances of lower toxicity than the compounds previously used.

**Figure 1:**
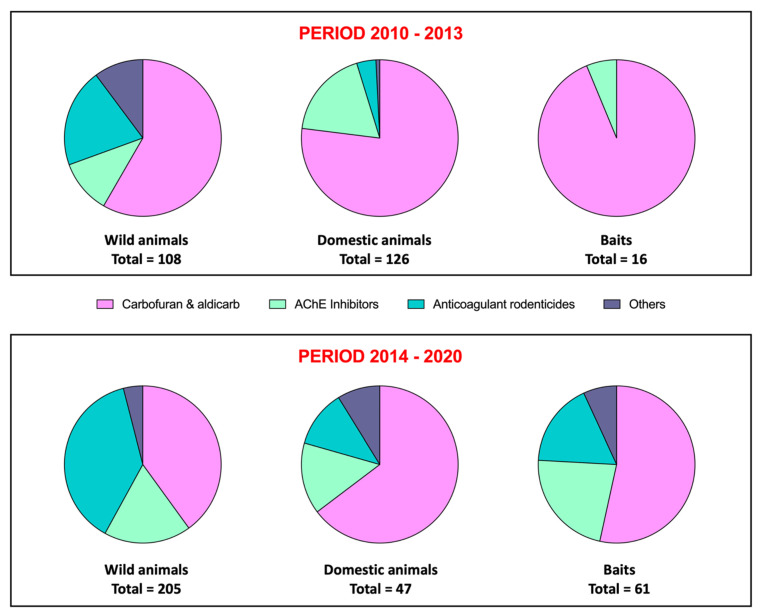
Comparison of the distribution of poisons involved in wildlife mortality cases grouped by type between the period 2010–2013 (upper panel) and the period 2014–2020 (lower panel).

In Figure 1, we present in graphical form a comparison of the distribution of poisons that have affected wild and domestic fauna in the Canary Islands in both study periods; the present study corresponds to the investigations after the regional law against the poison in natural areas came into force [34], as compared to our previous report, in which the cases were received quite informally [4]. A slight change in the pattern of use of poisons is evident, particularly regarding baits. Possibly, this indicates that in certain parts of the archipelago, carbofuran and aldicarb have been depleted, and mixtures of other compounds that are assumed to be less potent individually are beginning to be used.

**Figure 2:**
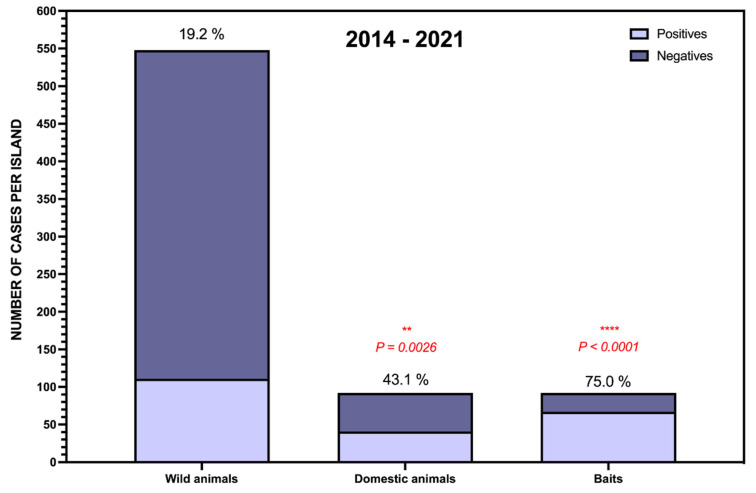
Proportion of positive cases among wild animals, domestic animals, and baits tested. ** *p* < 0.01; **** *p* < 0.0001.

With respect to the proportion of poison-positive cases among the total number of cases referred during this period, we found significant differences depending on whether they were wild or domestic animals. As shown in Figure 2, the percentage of positives among wildlife barely reached 20%, while in the case of dogs and cats, this percentage was more than double. This finding is not surprising, since it has been described that cats, and mainly dogs, when sick become much more visible to humans than wild animals, which, in these same circumstances, tend to seek refuge in their nests and burrows, and many of them are never found [46]. We highlight this fact because the cases of poison in wildlife could be much higher than what we have officially recorded from the samples submitted to us, because dying animals that hide are probably never found. As can be seen in the graph, in the baits analyzed, the percentage of positive identifications was very high (75%). This finding is also very logical, since when baits are sent to us, their appearance and location attract attention in most cases, and there are strong suspicions that they have been laced with poison, and virtually all these samples are submitted to the laboratory for investigation. Additionally, the poisons of interest are expected to be more concentrated and perhaps also more stable in the bait source compared to biological samples, so even if there is degradation, it is more likely that high enough concentrations will remain to allow detection.

During these years, the diagnosis of death by poisoning has been made based either on the comparison of the liver concentrations found with the data available in the literature, on the calculation of the toxic dose from the blood concentration and the apparent volume of distribution of the poison found (when these data were available), and on the clinical or presumptive findings of poisoning collected in the files by the environmental police agents or by the veterinarians who attended the animals. It was not always possible to attribute the death of the animal to the toxic substances found, which was either because the concentrations found did not seem sufficiently high or because the advanced state of degradation of the samples did not allow knowing if there was degradation of the chemical substances decreasing their concentration or because there were no reference data. Therefore, the doubtful cases have not been included as positive in this study. However, although the outcome does not change once a certain threshold concentration of a chemical is exceeded that is considered potentially lethal, we do consider that the finding of extremely high concentrations in liver would reflect exposure to massive doses of the poison, probably after ingestion of a bait, thus indirectly suggesting a pattern of intentionality in the poisoning. Obviously, intentionality cannot be inferred from concentrations alone, so we only state this as a working hypothesis based on our experience. According to this hypothesis, we wanted to compare whether there were also differences in the type of poisons affecting one or the other type of animal (wildlife vs. domestic) and the concentrations found for each of them in both types of animals (Table 2).

We found significant differences in the concentrations of some toxicants. Thus, the most striking case is that of aldicarb, which presented a median value about 40 times higher in the series of wildlife than in that of domestic animals (*p* < 0.05), with several animals of different incidents presenting massive concentrations of this substance in their liver. Something similar was observed with brodifacoum, which also presented significantly higher concentrations in the livers of wild animals than in domestic animals (*p* < 0.0001). In the first case, the use and even possession of aldicarb has been prohibited for almost 20 years [45], so that accidentality could be ruled out in all cases. In the case of brodifacoum, there is a limitation for its outdoor use in the agricultural environment, so this result was to some extent surprising. The high concentrations found in wild animals compared to dogs and cats probably suggests a pattern of intentionality, at least in part of the cases. Even more so considering that with another frequently detected rodenticide, bromadiolone, just the opposite is true. Bromadiolone levels were significantly higher in the livers of intoxicated pets (Table 2). This second-generation anticoagulant is a legal and very commonly used rodenticide, both in urban and rural areas, including agriculture and livestock. It is more than likely that most of the cases involving this toxicant, both in domestic and wild animals, are due to accidental poisoning, probably secondary to the ingestion of poisoned rodents, rather than to the ingestion of baits. With respect to the other chemical substance for which there was a significant difference between wild and domestic animals, permethrin, it should be noted that this is a compound of particularly high toxicity to cats [47], which is the species with the highest liver concentrations. It is true that the literature describes that some cat poisonings are due to the accidental application of flea products labeled for dogs. It might be reasonable for well-meaning people to apply permethrin-containing dog products to cats with the goal of helping rather than harming them. However, it should also be noted that permethrin was also identified in two of the baits analyzed, so, at least in some cases, the high concentrations found could also point to a pattern of intentionality aimed at eliminating stray cats. Finally, some compounds were only detected in wildlife specimens, such as amobarbital, dimethoate, fenamiphos, flocoumafen, and pirimiphos methyl, while others were only detected in domestic animals, such as alpha chloralose, chlorpyrifos, imidacloprid, methiocarb, and tetramethrin, although some of the chemicals were detected only sporadically. Among them, it is worth highlighting the detection of amobarbital, since it represents the first case of poisoning by barbiturates recorded in the Canary Islands, unlike what has been reported recently for mainland Spain, where barbiturates were involved in up to 3.4% of the poisonings detected [31].

**Figure 3:**
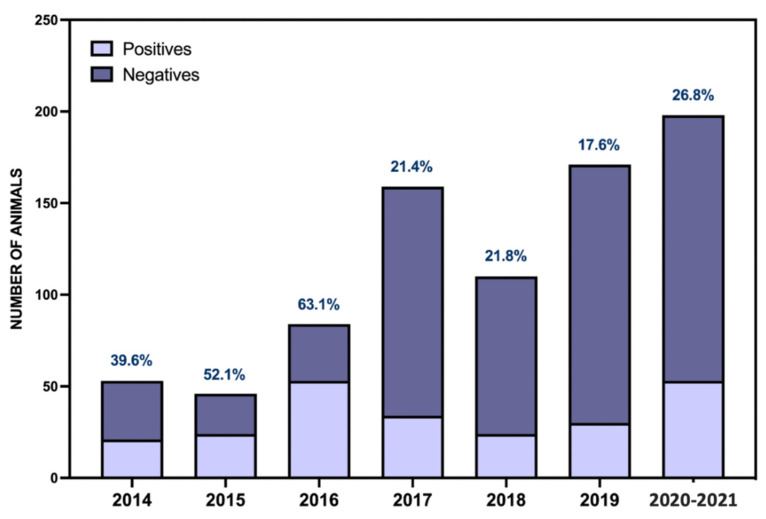
Temporal evolution of positive/negative cases for poisoning as cause of death.

During this period, the number of positive cases per year has not shown a downward trend (Figure 3), remaining stable. What has increased is the number of samples received in our laboratory to rule out cases of poisoning, as this increase has been very noticeable from 2017. We previously pointed out that one of the biggest problems in the investigation of wildlife poisoning is that a large part of the cases may never be detected due to the elusive behavior of most species when they are seriously ill [46]. Precisely because of this, and in the context of the Canarian strategy against poison [34], two canine patrols trained in the detection of poisons and carcasses have come into operation: the first one was on the island of Gran Canaria, which began operating in 2017, and the second one came into operation in 2020 and covers the islands of Fuerteventura and Lanzarote. As can be seen in Figure 3, these milestones coincide with respective increases in the receipt of samples and the corresponding increase in the detection of poisoning cases. Even so, although there was a quantitative increase in the number of positive cases identified, this did not alter the proportion of cases in relation to the total number of samples received in the laboratory, remaining approximately the same. Only in the last period, from January 2020 onwards, has there been a slight increase in the number of positive cases detected, but it is too early to conclude whether this trend will continue over time. Future studies will test whether canine patrols contribute effectively to the visibility of wildlife poisoning cases, as has been described in other regions [46].

**Figure 4:**
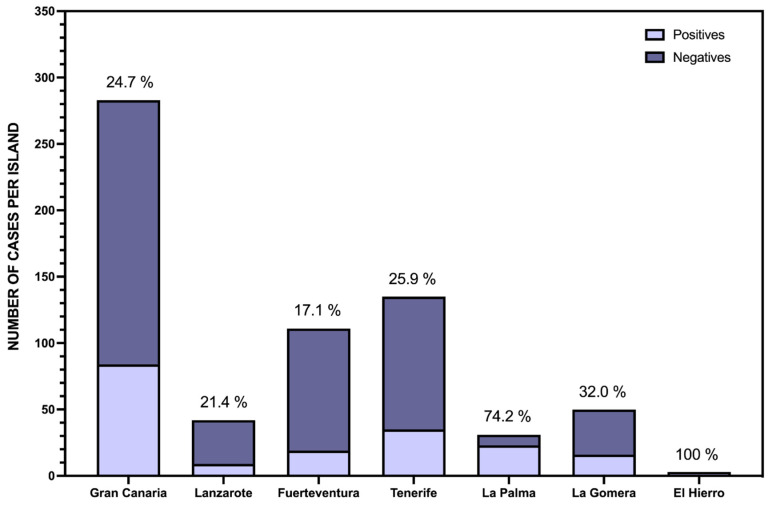
Distribution of poisoning cases diagnosed in relation to the total number of cases referred from each of the islands of the Canary archipelago. In the bar corresponding to Lanzarote, those from the island of La Graciosa have been included due to its administrative dependence on that island until 2018.

The Canary Islands include eight inhabited islands, five islets, eight rocks, and the sea. The animals received in our service correspond only with the eight inhabited islands, although the smallest of all, La Graciosa, is usually considered together with Lanzarote, on which it has depended administratively until very recently (2018). Figure 4 represents how the cases received have been distributed in relation to the island on which the incident occurred. As can be seen, most of the cases were recorded on the island of Gran Canaria, which is where our laboratory is physically located. Probably, since it is an archipelago, this is due to logistical reasons, since it was easier to send the samples, especially during the first few years of operation. It is also noteworthy that one of the canine patrols, the first to become operational, is also based on this island. Although this patrol operates throughout the archipelago, it is true that the highest rate of interventions occurs on the island of Gran Canaria, so this undoubtedly influences this difference with respect to the rest of the archipelago. From the islands of Fuerteventura and, to a much lesser extent, Lanzarote, a good number of cases have also been received, especially in the last two years, also coinciding with the entry into operation of the second canine patrol, which is physically located on the island of Fuerteventura. Our results indicate that dogs trained in the detection of poisons are a valuable aid in detecting this serious environmental problem. With respect to the cases coming from the islands farthest from our laboratory (La Gomera, La Palma, and El Hierro), the high percentage of positives is striking, reaching 100% of those sent from the island of El Hierro. From our point of view, this reflects the logistical problems that have existed during these first years of operation of the Canary Islands anti-poison strategy, which has meant that the cases sent for investigation from these islands have been meticulously selected. The case of the island of Tenerife is noteworthy, since it is the most populated island of the archipelago, the one with the greatest agricultural and livestock activity, and one of the islands with the greatest biodiversity. However, the low rate of samples investigated on this island is surprising. This situation will probably change significantly in the next few years, since a third canine patrol is expected to start operating, which will be located on this island, and which will undoubtedly help to make visible the cases of poisoning that we believe are going unnoticed there.

Finally, we wanted to study the main determinants of the pattern of poison use in the Canary Islands, using the available variables, mainly in relation to parameters related to the species, land use, and population density, as has been established in other research studies [21].

**Figure 5:**
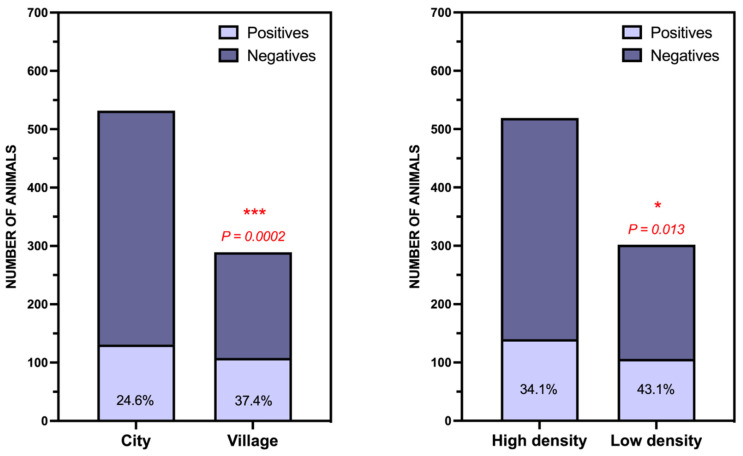
Study of the determinants of the incidence of wildlife poisoning in the Canary Islands. (Left) Type of locality according to the number of inhabitants (cut-off point = 19,657 inhabitants (median value)), (Right) Population density (cut-off point = 161 inhabitants/km^2^ (median value). * *p* < 0.05; *** *p* < 0.001.

First, we studied the influence of habitat (urban vs. rural), and population density (Figure 5). We found that the number of animals referred from cities is significantly higher than those referred from smaller towns (based on the number of inhabitants of the municipality in which the carcass was found, cut-off point = 19,657 inhabitants (median value)), but the percentage of positive cases is the inverse, being significantly higher in animals referred from rural localities (Figure 5, left). Something very similar occurred with the population density (cut-off point = 161 inhabitants/km^2^). The number of cases referred from less densely populated areas was lower, which was probably because it is more difficult to find the carcasses, but the percentage of positive cases among the animals referred from these areas (43.1%) was significantly higher than those referred from more populated areas (34.1%) (Figure 5, right). This finding seems quite logical to us, given that most of the chemical compounds we have detected are agricultural pesticides, so their availability in rural areas should be greater.

**Figure 6:**
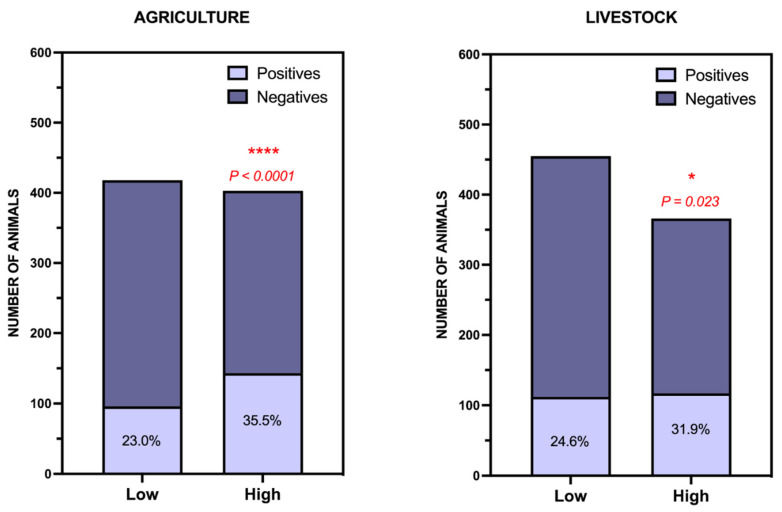
Study of the determinants of the incidence of wildlife poisoning in the Canary Islands. (Left) Influence of agricultural activity (cultivated area surface (%)) and (Right) Livestock influence (number of livestock per hectare). * *p* < 0.05; **** *p* < 0.0001.

We also explored the relationship with agricultural and livestock activity in the municipalities where the carcasses that were sent to our laboratory were found (Figure 6). First, with respect to agricultural activity, we found that there was a relationship, with the percentage of positive cases being significantly higher in those municipalities with greater agricultural activity. Several cut-off points were used to calculate this (number of cultivated hectares, cultivated area surface (%), and cultivated area per inhabitant), and with all of them, statistical significance was maintained (Figure 6, left). We also find this result logical for the same reason discussed above: the availability of agricultural products, whether permitted or not, is closely related to agriculture. According to our results, the same is true for livestock activity, since the situation is repeated: the percentage of positive cases in municipalities with more livestock is significantly higher than in those with less livestock activity. This significance was maintained for all the cut-off points tested (total number of livestock in the municipality; livestock density (number of heads/hectare); and livestock density/population density) (Figure 6, right).

In conjunction with our previous report of poisonings in the Canary Islands [4], our results indicate that the incidence of poisonings in this archipelago is very high and probably higher than in other European regions [7,9,14,41,48]. Moreover, the profile of toxicants that we have found suggests that many of these poisonings occur intentionally, given the high prevalence of substances whose use in agriculture would be illegal throughout the EU. Since this practice is highly detrimental to biodiversity, as well as a major public health problem, it is necessary that the authorities enact effective measures on the marketing of toxic chemicals, the control of stocks of banned chemicals, the implementation of educational programs and the effective criminal prosecution of poisoners to prevent, or at least minimize, the incidence of this harmful practice. From the literature, it can be assumed that the numbers reported in those studies only represent an approximation of the actual incidence of wildlife mortality, because it has been estimated that less than 10% of poisoning cases are detected and sent to a forensic laboratory [49]. This is especially relevant for wildlife because sick animals are often less visible and many die in nests, burrows, or inaccessible locations. The presence of canine patrols probably increases detection rates, as we have found in this study, but it is still quite likely that a good portion of cases will go undetected, particularly regarding wildlife. Whatever the case, our findings indicate that the actual incidence of poisoning mortality in the Canary Islands is very high and certainly higher than in other European regions.

We have observed a very slight decreasing trend in the use of prohibited substances, which is much lower than the progressively decreasing annual trend reported in other regions [50,51]. Carbofuran, aldicarb, and other banned AChE inhibitors were used extensively in agriculture in the Canary Islands, which are mainly associated with the cultivation of banana and other export vegetables. It is likely that there are still significant stocks of these banned substances on many farms, although there is also the possibility that they are still being acquired on the black market [50].

Although numerous measures have been taken to correct this problem in this region, it is probably too early to verify their efficacy. The authorities should take different measures to correct the circumstances that motivate the intentional poisoning of animals to curb, or at least minimize, this serious problem that seriously threatens biodiversity, animal welfare, and public health.

## Figures and Tables

**Table 1:** Identification of pesticides in animals and baits from poisoning episodes occurred in the Canary Islands during the period 2014–2020.

	Principal Toxicant (s)				
	Carbofuran	Aldicarb	Other AChE Inhibitors	Anticoag.	Others
Wild animals					
*Accipiter nisus*				1	
*Alectoris rufa*			1	1	
*Anas platyrrhynchos*	5				
*Ardea cinerea*					2
*Asio otus*	3		3	14	2
*Atelerix algirus*		1		1	
*Buteo buteo*	11	2	2	12	
*Chalcides simonyi*				1	1
*Columba livia*	7		18		
*Corvus corax*	14		7		1
*Pyrrhocorax pyrrhocorax barbatus*	7				
*Falco tinnunculus*	6		5	21	
*Gallotia galloti*	8	1	3	9	
*Larus michaellis*	1				
*Neophron percnopterus*	12		1	2	2
*Oryctolagus cuniculus*			2	2	
*Turdus merula*	3	2	5		
*Tyto alba*				2	1
Domestic animals					
Cats	11	2	4	4	
Dogs	9	6	6	2	3
Baits and suspicious materials					
Meat or feed	24	9	16	8	4

**Table 2:** Comparison of concentrations of chemicals identified in confirmed poisoning cases between wild and domestic animals.

	Wild Animals		Domestic Animals		
Chemical	Median	Range (p25–p75)	Median	Range (p25–p75)	*p*
Aldicarb	20,298.1 *	82.4–343,267.4	402.5	101.3–1913.2	0.0387
Alpha chloralose			3498.3		
Amobarbital	35,677				
Brodifacoum	1128.8 ****	585.6–3098.3	338.5	291.5–456.7	<0.0001
Bromadiolone	331.0	222.2–646.5	960.7 *	389.5–1902.1	0.0432
Carbofuran	1499.2	353.3–4412.7	3702.3	420.1–23,007.9	n.s.
Chlorpyrifos			8832.4		
Dimethoate	5589.2	1987.2–12,377.3			
Fenamiphos	7469.5				
Flocoumafen	1223.2	287.1–2298.6			
Imidacloprid			1034.5	877.6–3347.8	
Methiocarb			2336.5		
Methomyl	2155.6	788.3–21,887.3	2332.2	987.4–7886.5	n.s.
Oxamyl	556.8	344.3–12,443.8	766.3	677.8–8876.5	n.s.
Permethrin	381.2	227.4–1886.3	1500.2 *	998.4–5667.9	0.0234
Pirimiphos methyl	1223.4	556.7–3446.7			
Tetramethrin			921.6		

* *p* < 0.05; **** *p* < 0.0001

## Appendix A

**Table A1:** List of compounds analyzed together with their group and technique of analysis.

Nº	Compound	Group ^a^	Technique ^b^	Nº	Compound	Group ^a^	Technique ^b^
1	4,4′-Dichlorobenzophenone	Pesticides, OCPs	GC	156	Pencycuron	Pesticides	LC
2	4,4′-Dicofol	Pesticides, OCPs	GC	157	Pendimethalin	Pesticides	LC
3	Abamectine	Pesticides	LC	158	Permethrin	Pesticides	GC
4	Acephate	Pesticides	LC	159	Phosalone	Pesticides	LC
5	Acetamiprid	Pesticides	LC	160	Phosmet	Pesticides	LC
6	Acrinathrin	Pesticides	LC	161	Phosmet oxon	Pesticides	LC
7	Aldicarb	Pesticides	LC	162	Phthalimide (Folpet deg)	Pesticides	GC
8	Aldicarb sulfone	Pesticides	LC	163	Pirimicarb	Pesticides	LC
9	Atrazine	Pesticides	LC	164	Pirimiphos ethyl	Pesticides	LC
10	Azinphos methyl	Pesticides	LC	165	Pirimiphos methyl	Pesticides	LC
11	Azoxystrobin	Pesticides	LC	166	Prochloraz	Pesticides	LC
12	Benalaxyl	Pesticides	LC	167	Procymidone	Pesticides	GC
13	Bendiocarb	Pesticides	LC	168	Profenofos	Pesticides	LC
14	Bifenthrin	Pesticides	GC	169	Propargite	Pesticides	LC
15	Bitertanol	Pesticides	LC	170	Propiconazole		
16	Boscalid	Pesticides	GC	171	Propoxur	Pesticides	LC
17	Bromopropylate	Pesticides	GC	172	Propyzamide (pronamide)	Pesticides	LC
18	Bromuconazole	Pesticides	LC	173	Proquinazid	Pesticides	LC
19	Bupirimate	Pesticides	LC	174	Prothioconazole-desthio	Pesticides	LC
20	Buprofezin	Pesticides	LC	175	Prothiophos	Pesticides	GC
21	Cadusafos	Pesticides	LC	176	Pyraclostrobin	Pesticides	LC
22	Carbaryl	Pesticides	LC	177	Pyrazophos	Pesticides	LC
23	Carbofuran	Pesticides	LC	178	Pyridaben	Pesticides	LC
24	Carbofuran-3-hydroxy	Pesticides	LC	179	Pyridaphenthion	Pesticides	LC
25	Chlorantraniliprole	Pesticides	LC	180	Pyrimethanil	Pesticides	GC
26	Chlorfenapyr	Pesticides	GC	181	Pyriproxifen	Pesticides	LC
27	Chlorobenzilate	Pesticides	GC	182	Quinalphos	Pesticides	LC
28	Chlorfenvinphos	Pesticides	LC	183	Quinoxyfen	Pesticides	LC
29	Chlorpropham	Pesticides	GC	184	Rotenone	Pesticides	LC
30	Chlorpyrifos	Pesticides	GC	185	Simazine	Pesticides	LC
31	Chlorpyrifos methyl	Pesticides	GC	186	Spirodiclofen	Pesticides	LC
32	Chlorthal dimethyl	Pesticides	GC	187	Spiromesifen	Pesticides	LC
33	Clofentezine	Pesticides	LC	188	Spirotetramat	Pesticides	LC
34	Clothianidin	Pesticides	LC	189	Spirotetramat-enol	Pesticides	LC
35	Coumachlor	Pesticides	LC	190	Spiroxamine	Pesticides	GC
36	Coumaphos	Pesticides	LC	191	Tebuconazole	Pesticides	LC
37	Cyazofamid	Pesticides	LC	192	Tebufenocide	Pesticides	LC
38	Cyflufenamid	Pesticides	LC	193	Tebufenpyrad	Pesticides	LC
39	Cyfluthrin	Pesticides	GC	194	Teflubenzuron (artifact 3)	Pesticides	GC
40	Cyhalothrin (lambda isomer)	Pesticides	LC	195	Tefluthrin	Pesticides	GC
41	Cymoxanil	Pesticides	LC	196	Telodrin	Pesticides	GC
42	Cypermethrin	Pesticides	GC	197	Terbufos	Pesticides	GC
43	Cyproconazole	Pesticides	LC	198	Terbuthylazine	Pesticides	LC
44	Cyprodinil	Pesticides	GC	199	Tetrachlorvinphos	Pesticides	LC
45	Deltamethrin	Pesticides	GC	200	Tetraconazole	Pesticides	LC
46	Demeton-S-methyl	Pesticides	LC	201	Tetradifon	Pesticides	GC
47	Demeton-S-methyl-sulfone (Dioxydemeton)	Pesticides	LC	202	Tetramethrin	Pesticides	GC
48	Diazinon	Pesticides	GC	203	Thiacloprid	Pesticides	LC
49	Dichlofluanid	Pesticides	GC	204	Thiamethoxam	Pesticides	LC
50	Dichloran	Pesticides	GC	205	Thiodicarb	Pesticides	LC
51	Diethathyl ethyl	Pesticides	LC	206	Tolclofos methyl	Pesticides	GC
52	Diethofencarb	Pesticides	LC	207	Tolylfluanid	Pesticides	GC
53	Difenoconazole	Pesticides	LC	208	Triadimefon	Pesticides	LC
54	Diflubenzuron	Pesticides	LC	209	Triadimenol	Pesticides	LC
55	Diflufenican	Pesticides	LC	210	Triazophos (hostathion)	Pesticides	LC
56	Dimethenamide	Pesticides	LC	211	Trichlorfon	Pesticides	LC
57	Dimethoate	Pesticides	LC	212	Trifloxystrobin	Pesticides	LC
58	Dimethomorph	Pesticides	LC	213	Triflumizole	Pesticides	LC
59	Diniconazole-M	Pesticides	LC	214	Triflumuron	Pesticides	LC
60	Dinocap	Pesticides	LC	215	Trifluralin	Pesticides	GC
61	Diphenylamine	Pesticides	LC	216	Triticonazole	Pesticides	LC
62	Endosulfan alfa	Pesticides, OCPs	GC	217	Vinclozolin	Pesticides	GC
63	Endosulfan beta	Pesticides, OCPs	GC	218	Zoxamide	Pesticides	LC
64	EPN	Pesticides	LC	219	Aldrin	OCPs	GC
65	Epoxiconazole	Pesticides	LC	220	Dichlorodiphenyldichloroethane (p,p’ DDD)	OCPs	GC
66	Esfenvalerate	Pesticides	GC	221	Dichlorodiphenyldichloroethylene (p,p’ DDE)	OCPs	GC
67	Ethion	Pesticides	LC	222	Dieldrin	OCPs	GC
68	Ethofumesate	Pesticides	GC	223	Endrin	OCPs	GC
69	Ethoprophos	Pesticides	LC	224	Heptachlor	OCPs	GC
70	Etofenprox	Pesticides	LC	225	Hexachlorobenzene	OCPs	GC
71	Etoxazole	Pesticides	LC	226	Hexachlorocyclohexane (alpha)	OCPs	GC
72	Famoxadone	Pesticides	LC	227	Hexachlorocyclohexane (gamma, lindane)	OCPs	GC
73	Fenamidone	Pesticides	LC	228	Hexachlorocyclohexano (beta)	OCPs	GC
74	Fenamiphos	Pesticides	LC	229	Hexaclorociclohexano (delta)	OCPs	GC
75	Fenamiphos sulfone	Pesticides	LC	230	Mirex	OCPs	GC
76	Fenamiphos sulfoxide	Pesticides	LC	231	PCB 28	PCBs	GC
77	Fenarimol	Pesticides	GC	232	PCB 52	PCBs	GC
78	Fenazaquin	Pesticides	LC	233	PCB 77	PCBs	GC
79	Fenbuconazole	Pesticides	LC	234	PCB 81	PCBs	GC
80	Fenbutatin oxide	Pesticides	LC	235	PCB 101	PCBs	GC
81	Fenitrothion	Pesticides	GC	236	PCB 105	PCBs	GC
82	Fenoxycarb	Pesticides	LC	237	PCB 114	PCBs	GC
83	Fenpropathrin	Pesticides	LC	238	PCB 118	PCBs	GC
84	Fenpropimorph	Pesticides	LC	239	PCB 123	PCBs	GC
85	Fenpyroximate	Pesticides	LC	240	PCB 126	PCBs	GC
86	Fenthion	Pesticides	LC	241	PCB 138	PCBs	GC
87	Fenthion oxon	Pesticides	LC	242	PCB 153	PCBs	GC
88	Fenthion oxon sulfone	Pesticides	LC	243	PCB 156	PCBs	GC
89	Fenthion oxon sulfoxide	Pesticides	LC	244	PCB 157	PCBs	GC
90	Fenthion sulfone	Pesticides	LC	245	PCB 167	PCBs	GC
91	Fenthion sulfoxide	Pesticides	LC	246	PCB 169	PCBs	GC
92	Fenvalerate	Pesticides	GC	247	PCB 180	PCBs	GC
93	Fipronil	Pesticides	LC	248	PCB 189	PCBs	GC
94	Fipronil sulfide	Pesticides	GC	249	PBDE 28	PBDEs	GC
95	Fluazinam	Pesticides	LC	250	PBDE 47	PBDEs	GC
96	Flubendiamide	Pesticides	LC	251	PBDE 85	PBDEs	GC
97	Flucythrinate	Pesticides	GC	252	PBDE 99	PBDEs	GC
98	Fludioxonil	Pesticides	LC	253	PBDE 100	PBDEs	GC
99	Flufenoxuron	Pesticides	LC	254	PBDE 153	PBDEs	GC
100	Fluopyram	Pesticides	LC	255	PBDE 154	PBDEs	GC
101	Fluquinconazole	Pesticides	LC	256	PBDE 183	PBDEs	GC
102	Flusilazole	Pesticides	LC	257	Acenaphthene	PAHs	GC
103	Flutolanil	Pesticides	LC	258	Acenaphthylene	PAHs	GC
104	Flutriafol	Pesticides	LC	259	Anthracene	PAHs	GC
105	Fluvalinate tau	Pesticides	LC	260	Benzo[a]anthracene	PAHs	GC
106	Fonofos	Pesticides	GC	261	Benzo[b]fluoranthene	PAHs	GC
107	Fosthiazate	Pesticides	LC	262	Chrysene	PAHs	GC
108	Hexaconazole	Pesticides	LC	263	Fluoranthene	PAHs	GC
109	Hexaflumuron	Pesticides	LC	264	Fluorene	PAHs	GC
110	Hexythiazox	Pesticides	LC	265	Naphthalene	PAHs	GC
111	Imidacloprid	Pesticides	LC	266	Phenanthrene	PAHs	GC
112	Indoxacarb	Pesticides	LC	267	Pyrene	PAHs	GC
113	Iprodione	Pesticides	GC	268	Brodifacoum	ARs	LC
114	Iprovalicarb	Pesticides	LC	269	Bromadiolone	ARs	LC
115	Isocarbophos	Pesticides	GC	270	Coumatetralyl	ARs	LC
116	Isofenphos methyl	Pesticides	LC	271	Difenacoum	ARs	LC
117	Isoprothiolane	Pesticides	LC	272	Difetihalone	ARs	LC
118	Kresoxim methyl	Pesticides	LC	273	Flocoumafen	ARs	LC
119	Linuron	Pesticides	LC	274	Warfarin	ARs	LC
120	Lufenuron	Pesticides	LC	275	Albendazole	PhACs	LC
121	Malaoxon	Pesticides	LC	276	Cefuroxima axetil	PhACs	LC
122	Malathion	Pesticides	LC	277	Chloramphenicol	PhACs	LC
123	Mandipropamid	Pesticides	LC	278	Cloxacillin	PhACs	LC
124	Mefenoxam (metalaxyl-M)	Pesticides	LC	279	Cortiscosterone 21 acetate	PhACs	LC
125	Mepanipyrim	Pesticides	LC	280	Dexamethasone	PhACs	LC
126	Metaflumizone	Pesticides	LC	281	Diclofenac	PhACs	LC
127	Metalaxyl	Pesticides	GC	282	Eprinomectin	PhACs	LC
128	Metaldehyde	Pesticides	LC	283	Fenbendazole	PhACs	LC
129	Metconazole	Pesticides	LC	284	Flunixin	PhACs	LC
130	Methamidophos	Pesticides	LC	285	Imipenem	PhACs	LC
131	Methidathion	Pesticides	LC	286	Josamycin	PhACs	LC
132	Methiocarb	Pesticides	LC	287	Ketoprofen	PhACs	LC
133	Methiocarb sulfone	Pesticides	LC	288	Mebendazole	PhACs	LC
134	Methiocarb sulfoxide	Pesticides	LC	289	Mefenamic acid	PhACs	LC
135	Methomyl	Pesticides	LC	290	Metronidazole	PhACs	LC
136	Methomyl oxime	Pesticides	LC	291	Moxidectin	PhACs	LC
137	Methoxyfenozide	Pesticides	LC	292	Naproxen	PhACs	LC
138	Metrafenone	Pesticides	LC	293	Oxfendazole	PhACs	LC
139	Mevinphos (phosdrin)	Pesticides	LC	294	Penicilina V	PhACs	LC
140	Monocrotophos	Pesticides	LC	295	Sulfacetamide	PhACs	LC
141	Myclobutanil	Pesticides	LC	296	Sulfacloropiridacine	PhACs	LC
142	N,N-Dimethyl-N’-p-tolylsulphamide (DMST)	Pesticides	LC	297	Sulfadiacine	PhACs	LC
143	N,N-dimethylformamidine (DMF)	Pesticides	LC	298	Sulfadimetoxine	PhACs	LC
144	Nuarimol	Pesticides	LC	299	Sulfadoxine	PhACs	LC
145	Ofurace	Pesticides	LC	300	Sulfameracine	PhACs	LC
146	Omethoate	Pesticides	LC	301	Sulfametacine	PhACs	LC
147	Oxadixyl	Pesticides	LC	302	Sulfametizole	PhACs	LC
148	Oxamyl	Pesticides	LC	303	Sulfametoxazole	PhACs	LC
149	Oxamyl oxime	Pesticides	LC	304	Sulfametoxipiridacine	PhACs	LC
150	Oxyfluorfen	Pesticides	GC	305	Sulfamonomethoxine	PhACs	LC
151	Paclobutrazol	Pesticides	LC	306	Sulfanilamide	PhACs	LC
152	Paraoxon methyl	Pesticides	GC	307	Sulfapiridine	PhACs	LC
153	Parathion ethyl	Pesticides	GC	308	Sulfaquinoxaline	PhACs	LC
154	Parathion methyl	Pesticides	GC	309	Sulfisoxazole	PhACs	LC
155	Penconazole	Pesticides	LC	310	Tolfenamic acid	PhACs	LC

^a^ PBDE—Polybrominated diphenyl ethers, OCP—Organochlorine pesticides, PAH—Polycyclic aromatic hydrocarbon, PCB—Polychlorinated biphenyl, PhACs—Pharmaceuticals Active Compounds, Ars—Anticoagulant Rodenticides, P-IS—Procedural Internal Standard. ^b^ Gas chromatography (GC) or liquid chromatography (LC), both coupled with tandem triple quadrupole mass spectrometry.
